# Enhancing capacity for clinical practice guidelines in South Africa

**DOI:** 10.11604/pamj.2020.36.18.20800

**Published:** 2020-05-13

**Authors:** Taryn Young, Janine Dizon, Tamara Kredo, Michael McCaul, Eleanor Ochodo, Karen Grimmer, Quinette Louw

**Affiliations:** 1 Centre for Evidence-based Health Care, Division of Epidemiology and Biostatistics, Faculty of Medicine and Health Sciences, Stellenbosch University, PO Box 241, Cape Town, 8000, South Africa; 2 International Centre for Allied Health Evidence (iCAHE), City East Campus, P4-18 North Terrace, University of South Australia, Adelaide 5000, Australia; 3 Cochrane South Africa, South African Medical Research Council, P.O. Box 19070, Tygerberg, 7505, South Africa; 4 Division of Clinical Pharmacology, Department of Medicine, Faculty of Medicine and Health Sciences, Stellenbosch University, Cape Town, South Africa; 5 Physiotherapy Division, Faculty of Medicine and Health Sciences, Stellenbosch University, Cape Town, South Africa; 6 Clinical Education and Training, VITA, Flinders University, Bedford Park, Adelaide, SA 5042

**Keywords:** Clinical practice guideline, capacity development, evaluation

## Abstract

**Introduction:**

Use of good quality, evidence-informed and up-to-date clinical practice guidelines (CPGs) has the potential to impact health outcomes. This paper describes the development, implementation and evaluation of a dedicated CPG training course to address the training needs of CPG stakeholders in South Africa.

**Methods:**

We first reviewed the content and teaching strategies of existing CPG courses. This review consisted of a systematic review of teaching and learning strategies for guideline teams and a document review of existing courses offered by international guideline groups, universities and professional groups. We then strengthened an existing CPG course and evaluated it.

**Results:**

We found no studies on teaching and learning strategies for guideline teams. We identified six CPG courses being offered as full courses (part of a postgraduate degree program) by universities or as independent training for continuing professional education by professional groups. Contents focused on new guideline development. One course included alternative methods of guideline approaches such as contextualization and adaptation. The format varied from face-to-face sessions, to online sessions, group exercises and discussions, seminar format and project based activities. The revised CPG four-month long course that we implemented was designed to be pragmatic, reflective and contextually relevant. It used local guideline examples, authentic tasks, and an online forum for discussions and resources. It covered de novo CPG development, alternative methods of development (adopting, contextualising, adapting), and implementing CPGs. Course evaluation identified strengths and areas for improvement.

**Conclusion:**

Dedicated capacity development has potential to positively influence CPG development and implementation.

## Introduction

High quality, evidence-informed clinical practice guidelines (CPGs) offer a way of bridging the gap between policy, best-practice, local contexts and patient choice [1]. Good quality CPGs offer a ‘one stop shop’ for end-users [2]. They present current best evidence (primary, secondary or opinion-based) to answer questions pertaining to care for a health condition [3]. The development of CPGs has traditionally been motivated by the need to improve efficiency and cost-effectiveness of health system utilization, and to decrease costly and preventable mistakes and adverse events [4]. CPGs are intended to provide transparent synthesis of the evidence on which sound, ‘on balance’ judgments can be made by clinicians, administrators, policy-makers and patients. Evidence-informed decisions should minimise over-, under- or mis-use of services [5]. Constructing a good quality CPG and presenting recommendations in a user-friendly format is a more complex process, compared to constructing other forms of secondary evidence (such as systematic literature reviews) [6]. CPG construction usually involves multiple stakeholders including content experts, methodologists, systematic reviewers and guideline users. CPG methodologists have knowledge and experience to conduct evidence syntheses of multiple clinical questions for a single guideline, and guide the transparent and justified approach to assess the level of evidence to inform the final recommendation. Furthermore, the ability to formulate real-world recommendations is not only based on the evidence but also considers other factors such as applicability and acceptability to a local context. Consequently, specific training is required to understand the nuances of CPG writing, and to assist in efficient task completion.

South Africa has a long history of developing various CPGs. Key role players include the National Department of Health, professional societies, private sector and non-governmental organisations producing guidance for their respective constituents. Our team came together in 2014 to conduct the South African Guidelines Excellence (SAGE) Project [7]. We undertook a range of projects to understand the context, processes, and the need for, South African CPGs. Project SAGE explored perspectives of over 100 CPG stakeholders, regarding CPG development and use, how they assessed CPG believability and impact, how they implemented CPGs and what implementation barriers they faced [8-10]. Stakeholder interviews, particularly those involved with CPG development activities, consistently expressed a need for greater technical capacity to develop and implement CPGs. This was perceived to be important to ensure efficient and competent CPG construction, interpretation, updating and implementation. This finding was supported by the quantitative evaluation of 16 South African primary care clinical CPGs which were critically appraised for methodological quality. This activity found these CPGs to be substantially lacking in their reporting on methodological rigour, applicability and editorial independence [11, 12]. Project SAGE findings led to an innovative model of CPG transferability and implementation (adopt, contextualise, adapt) [8, 13]. In response to the gaps we identified in CPG reporting, and the expressed need for capacity building in evidence-informed CPG development and implementation, this paper describes the development, implementation and evaluation of a dedicated CPG training course to address the training needs of CPG stakeholders in South Africa.

## Methods

We first reviewed the content and teaching strategies of existing CPG courses using a systematic review of teaching and learning strategies for guideline teams and a document review of existing courses offered by international guideline groups, universities and professional groups. We then developed, implemented and evaluated a CPG course targeted to the needs of end-users in resource constrained environments.

**Systematic review:** to evaluate the effectiveness of teaching or learning strategies for CPGs, we searched for randomised and non-randomised trials, pre-post experimental studies or primary observational studies. We excluded studies that focussed only on methods for teaching evidence-based practice, and did not include CPGs. Our target participants included CPG writers, methodologists, implementers and evaluators. We developed a comprehensive strategy (Supplementary file 1) to search for all eligible studies available up to 29th November 2017, regardless of language or publication status. For published literature we searched Medline, Cochrane Database of Systematic Reviews (Effective Practice and Organization Group), Database of Abstracts of Reviews of Effects (DARE), Cumulative Index to Nursing and Allied Health Literature (CINAHL), current controlled trials, Educational Resources Information Center (ERIC) and Turning Research into Practice (Trip) database. We used the following search terms and tailored them appropriately to the different databases: ‘guide*’, ‘clinical practice guideline’, ‘teach*’, ‘learn*’. Three reviewers (EO, JD and VL) independently screened titles, abstracts and full texts of potentially eligible articles. To access unpublished literature, we searched websites of 17 guideline groups using a Google scoping search. We also contacted relevant experts in the field of CPGs. We aimed to conduct independent data extraction and risk of bias assessments and then to look at within group differences focusing on outcomes measured between baseline and post training. These outcomes could include short-term measures on teaching methods or programs (i.e. educational outcomes including feedback, evaluation, examination or self-report scores) and longer-term measures on the quality of the guideline process from development to evaluation.

**Document review of existing CPG courses:** we searched the internet for existing CPG courses using search term “*clinical practice guidelines courses” OR “guideline courses*”. We searched guideline sites and professional associations/organizations. We contacted 21 content experts via email, professional groups and the networks of Cochrane South Africa, Centre for Evidence Based Health Care and International Centre for Allied Health Evidence (iCAHE) to seek information regarding any CPG course training being offered, and for any evaluation of CPG courses delivered. Eighteen groups responded and we obtained the information from the websites of three groups who did not respond. We collected all possible information about CPG courses and extracted relevant information: *course description, objectives, content, teaching and learning strategies, mode of delivery, format, duration, outcomes and evaluation/assessments*. We used a descriptive qualitative framework to guide our data collection. Qualitative descriptive approaches are particularly useful in program assessment and development. We used content analysis to synthesize our data [14] regarding CPG course information based from the available data. Content analysis is an approach to analyzing findings from the document search by categorizing and classifying information to summarize the findings. Content analysis is the recommended synthesis approach for descriptive qualitative information. Findings were summarized on the basis of the data extracted.

**CPG course development and implementation:** we built on an existing master's level elective course on CPGs developed at Stellenbosch University, South Africa, some five years earlier. The original course was initially offered in 2009 as part of the MSc in Clinical Epidemiology. Between its inception and 2014 it had already shifted from being a mostly theory-based half-semester course to a practical full semester (4-month) course. However, with funding support from Project SAGE, together with reflection on elements of research and feedback from the project, we recognised that the needs of students working in resource constrained settings differ from those in other contexts. Thus, the course needed to be tailored to their needs. In 2015, the course was redesigned. Course characteristics are presented in Table 1. The course was offered in 2016 and a formal independent evaluation was conducted.

**Table 1:** CPG module characteristics

Name of module	Clinical Practice Guidelines
Aim	To enable participants to understand the different guideline approaches (de novo development and alternative methods), implementation, monitoring and evaluation of evidence based clinical practice guidelines
Objectives	1. Outline principles of evidence-based healthcare and study designs 2. Describe principles and different methods of evidence-based clinical-guideline development (de novo and adapting CPGs) 3. Critically appraise CPGs 4. Outline principles of grading the quality of evidence to inform CPG development 5. Discuss and apply evidence to decision frameworks for transparent evidence-informed CPG recommendations 6. Outline concepts in writing recommendations 7. Discuss principles of implementation of CPGs including consideration of stakeholders, and barriers and facilitators to successful implementation 8. Develop a plan for implementation of a CPG using appropriate strategies 9. Outline methods of monitoring and evaluating a CPGs
Content	Evidence-based Health care principles, principles of CPG development and appraisal, guideline development and the Evidence to Decision Framework, alternative methods of guideline development and implementing, and monitoring and evaluation CPGs
Learning approach	Adult blended learning (both online and face-to-face)
Assessment	Formative: Online assessments, forum discussions and authentic tasks (such as take home assignments) Summative: Portfolio of evidence
Entry requirements	Fundamentals of epidemiology and biostatistics
Short course certificate options	Competency or attendance

**CPG course evaluation:** a document review, observation of training delivered during the contact sessions and interviews with course participants were done. It evaluated whether the course material addressed the aim of the course and whether the predicted learning outcomes would be met; whether resources were appropriate to assist students to achieve the learning objectives; how assessments mapped to the learning outcomes; how well the teaching and learning approach mapped to best practice pedagogy; and determined the level of satisfaction of students with the teaching and learning processes of the course. All participants of 2016 were invited to participate. All course outlines and teaching material were provided to the independent evaluation team. They ascertained whether the course material addressed the course objectives, if the presentation of the course material was appropriate for the time-frame for the intensive, five day teaching mode and whether assessments mapped to the learning outcomes. In addition, the content expert ascertained whether the predicted learning outcomes were met by the teaching material. A pre-course demographics questionnaire was designed and emailed to all course participants one week prior to commencement of the face-to-face contact session in March (Supplementary file 2). This questionnaire was used to obtain: demographics (gender, name, age and current employment, contact details, citizenship); academic qualifications (and institution where the training was obtained); CPG training (whether students had received any prior CPG training, and if so, details about the training including where the training was obtained, the duration of the training program and brief description of the course content); and experience with CPG activity (any involvement with the CPG development, implementation of CPGs to the local context, or training related to CPGs). The workshop evaluation form was informed by the training evaluation framework. It focused on generic training issues and also explored the three key learning areas from each of the five days of face-to-face training.

The evaluation team observed training sessions and conducted post-course telephonic interviews one month after the March face-to-face contact sessions. The semi-structured telephonic interviews (in English, Supplementary file 3) focused on perceived change in knowledge or attitudes towards CPGs, barriers that participants anticipated in putting their learning outcomes into action, support for students post-completion so that barriers can be addressed, whether the course content information was considered comprehensive and value for money or time, and the level of satisfaction with the teaching and learning process. The evaluation team also interviewed an international content expert in the field (Supplementary file 4 for interview guide). A digital voice recorder was used to record interviews. Interviews lasted between thirty and sixty minutes. Interviews were transcribed verbatim. Names of participants did not appear on the transcriptions. The team used triangulation of data sources (interviews and workshop evaluations) and carried out inter-rater reliability checks on at least two of the observation days. Qualitative data from telephonic interviews, the observation schedule and the text generated from workshop evaluation were stored and managed using Atlas.ti. Inductive thematic content analysis was undertaken to analyse the interviews and identify key themes and sub-themes from the interview transcripts. Ethical clearance was obtained from the Health Research Ethics Committee (HREC), Stellenbosch University (ethics number N14/02/008A). All participants provided informed written consent.

## Results

**Figure 1:**
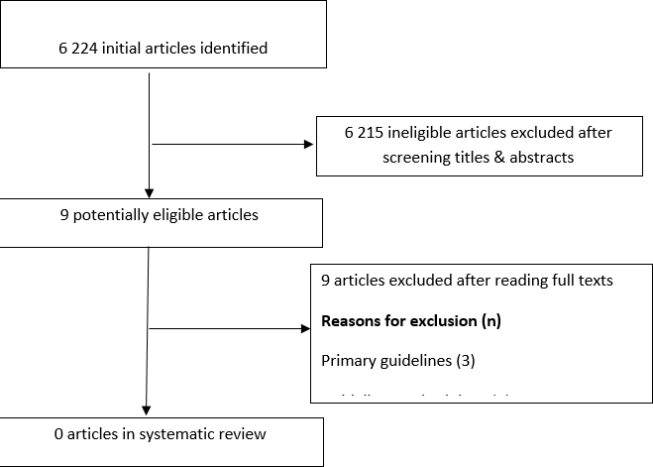
Flow chart of systematic review search results

Systematic review: our search for teaching and learning strategies yielded 6224 hits. Upon screening the titles and abstracts, we found nine potentially-eligible studies and excluded all upon reading the full texts, three articles were primary guidelines [15-17], two focused on guideline methodology [18, 19], two were on guideline implementation [20, 21] and two were surveys on methodological process [22, 23] (Figure 1). Thus, we found no eligible studies from our systematic literature review.

Document review of existing CPG courses: we found six (6) CPG courses, four (4) from universities (University of Sydney, McMaster University, Stellenbosch University, University of Toronto) and two (2) from professional groups (American Academy of Orthopaedic Surgeons, Kidney Health Australia) (Table 2). These were either offered by universities as a full course (part of a postgraduate degree program) or as independent training for continuing professional education by professional groups. The CPG courses focused on covering one or more components of the guideline development process (e.g. critical appraisal of guidelines, developing recommendations) as well as developing implementation plans (Table 2). One university-based CPG course included involvement of consumers in guidelines and examination of hospital-and community based guidelines (University of Sydney). Another university-based CPG course included other methods of guideline approaches such as contextualization and adaptation (Stellenbosch University) (Table 2). The format and delivery of CPG courses varied from face-to-face sessions, to online sessions, group exercises and discussions, seminar format and project based activities (Table 2). Courses ranged from two hours (short course) to 120 hours (full course program) depending on the content being covered. Only CPG courses offered by universities reported evaluations of their courses. Evaluation procedures included appraisal and barriers assessment, summative assessments, quizzes, examinations and online discussions (Table 2). In summary, it appeared from our comprehensive systematic search that CPG courses were offered by only a few universities and health professional groups. The focus of the courses was mainly on developing de novo CPGs or on one or more components of the process of developing de novo CPGs.

**Table 2:** Document review: summary of CPG courses offered by universities and professional organizations

Institution ac	Course title	Course objectives	Course contents	Course format	Course duration
University of Sydney http://sydney.edu.au/courses/uos/CEPI5306.	Clinical Practice Guidelines	No information available	Guideline development; critical appraisal of guidelines; introduction to implementation and evaluation of guidelines; involvement of consumers in guidelines; examination of hospital-based and community-based CPGs.	Classes offered online	No information available
McMaster University http://ebm.mcmaster.ca/course_materials/documents/family_medicine/2015/2015-Clinical-Practice-Guidelines-Module.pdf	Clinical practice guidelines	To learn to how to critically appraise a paper on clinical practice guidelines and incorporate them into your practice	Reading a clinical scenario given and formulating clinical decision; Reading an article and determining whether results of the paper apply in practice	No information available	No information available
Stellenbosch University http://www.sun.ac.za/english/faculty/healthsciences/Community%20Health/Documents/MSc_in_Clinical_Epidemiology_Course_outline_2015.pdf	Clinical Guidelines	To teach steps in developing, contextualizing and adapting CPGs, critical appraisal and implementation of CPGs	Principles of evidence-based clinical guidelines and guideline development; Moving from evidence to recommendations; Adapting, contextualizing guidelines Implementation of guidelines, guideline tools implementation strategies	Face-face 3-day block; on-line session; Reading; discussion	120 hours
University of Toronto http://ihpme.utoronto.ca/academics/rd/cehcr-mscphd/handbook/course-descriptions/#5305	Evidence-Based Guidelines	To understand characteristics of high-quality guidelines; To develop analytic framework to guide evidence extraction and synthesis; To discuss criteria for grading quality of evidence of diagnostic tests and interventions; To understand strength of recommendations; To develop skills in forming recommendations based on strength of evidence	Each student select a guideline topic applicable to their field and apply principles learned during seminars to the development of the guideline.	Seminar, interactive & project-based	m session per week
American Academy of Orthopedic Surgeons http://www.aaos.org/CustomTemplates/Content.aspx?id=6393	Developing an Evidence-Based CPG module	No information available	Clinical Guidelines Overview; Role, Structure, and Development of AAOS Guidelines; Phase 1: Simulated Recommendations; Phase 2: Inclusion Criteria; Phase 3: Literature Research: Finding Studies	No information available	**2 hours**
Kidney Health Australia http://www.cari.org.au/kha-cari_overview.html					

CPG course implementation and evaluation: the CPG four month long course was delivered at Stellenbosch University in 2016. The course was designed to be pragmatic, reflective and contextually relevant, using clinically relevant issues and examples, authentic tasks, online discussions and existing online resources. Students attended a 5-day teaching block with the remaining time spent in online engagement, self-study, readings and assessments. The course covered principles of both de novo CPG development and alternative methods of CPG writing (adopting, contextualising, adapting and updating CPGs). Students were assessed using online activities and written assignments followed by a summative portfolio of evidence which included reflective writing. The evaluation identified strengths and areas for improvement. Eighteen of the twenty one students who attended the course completed the demographic survey (Table 3). Most students were from South Africa, the majority had no previous training in writing CPGs and about a third had previous experience with using CPGs. Students rated the course highly, expressed that for most the course met their expectations, and that the content was relevant and they had learnt new things.

**Table 3:** Evaluating student's demographic and past CPG exposure

		Female	Male	Total
Educational Qualification	Bachelor's Degree	11	3	14
	Master's Degree	0	2	2
	PhD	2	0	2
Previous Training in CPGs	No Previous Training	13	4	17
	Some Previous Training	0	1	1
Experience with CPG Activity	No Previous Experience	9	3	12
	Some Previous Experience	4	2	6
Nationality	Cameroonian	1	0	1
	Congolese	4	0	4
	Kenyan	1	0	1
	Nigerian	0	1	1
	South African	6	2	8
	Zimbabwean	1	2	3

‘*I learnt about new sources of guidelines new work platforms to find guidelines. I also learnt about the other types of guidelines that are out there. The type of guideline I use are WHO guidelines so it's nice to learn about the other types of guidelines that are out there.’ ‘Each one [module] had unique aspects that is going to help me in my job. We had modules on phrasing questions we had modules on searching and finding guidelines which is what I do every day. We had modules on evaluating guidelines, we had modules on implementation the implementation part, maybe not so much, but it is something I am actually getting into it. They were all very helpful, and I'm going to be using the knowledge I have learnt in my everyday work actually*.’

Students appreciated the various forms of engagement, level of expertise and preparation by facilitators. They liked the use of practical exercises alongside didactic teaching, and rated the course as providing high value to their work. Students found the assessment process relevant, fair and appropriate. Some of the students gave recommendations for areas of improvement for future contact sessions-availability of materials before the block contact session, allotting more time for some sessions, early briefing on the assessment structure as well as provision of in-class notes. They also indicated some concern about having too much information covered during the 5-day teaching block.

‘*I wish they could have structured it in a way that you speak for an hour, somebody else comes for an hour, and you come back so that you have different people speaking. And they have more interactive slides and you ask a few questions, somebody answers. Now some of the people all they do is sit there in front of us and go through it so fast and you are more confused by the end of the hour than you were before*.’

The CPG content expert reviewer emphasized the importance of conveying concepts simply and ensuring adequate time for practical examples and reflection. He suggested additional relevant content such as the project management and chairing skills for CPG development as he described that ‘*at the heart of this process is a very mucky set of human interactions which can be managed well and can be managed badly, and can make a huge difference to the outcome*.’

## Discussion

With the growth in evidence-informed practices in the African region [24,25] and the recognised role of evidence-informed CPGs in shaping healthcare practices, there is an increasing need, and demand for, building capacity for evidence-informed CPG development and implementation [9, 10]. This paper described the development, implementation and evaluation of a dedicated CPG training course designed to meet specific development needs in South Africa and other similar resource constrained settings. The paucity of courses may be that CPG activities are traditionally learned on the job with panels often consisting of practicing clinical experts. However, this approach has long been recognised as insufficient [26]. Evidence of poor CPG quality, globally and in our region, provide a sound case for the need for additional training [12,27-29]. In response to these needs, we used an already available CPG course offered as part of the Masters in Clinical Epidemiology degree at Stellenbosch University [30], we transformed the content of this course to be more contextually relevant, pragmatic and student-focused. It covered de novo CPG development, alternative methods of writing (adopting, contextualising, adapting) as well as implementing CPGs. It used a blended learning approach, with both face-to-face and online sessions, coupled with assessment. It used local CPG examples, and authentic tasks. Course evaluation identified strengths and areas for improvement.

The course fills a specific gap for decision-makers from resource constrained settings as we share best practice knowledge for alternative development of CPGs. De novo CPG development is time and resource intensive requiring technical skills for conducting systematic reviews, funding to host meetings and ensure participation and consultation as broadly as possible. For this reason, alternative methods have been developed and our course is unique in generating awareness and discussion of these approaches [13, 31]. We are already seeing the benefits of skills development as students exposed to CPG training have lead CPG processes using rigorous reportable methods [32, 33]. Despite the demand for improving CPG-related skills, particularly in countries with scarce healthcare and training resources, there are limited robust evaluations of CPG capacity-development initiatives. The findings of our systematic assessment of existing international CPG training courses underpinned a targeted comprehensive CPG training programme. The course evaluation followed good practice methods for teaching evaluation including a review of documents, observation, interviews with faculty, and a survey of students. The inclusion of independent evaluation team, enhanced the objective collection of data from students. The evaluation had limitations as it did not assess the longer term effectiveness of learning to determine whether learning outcomes were achieved. Despite these limitations, this process could inform future capacity development initiatives in evidence production and uptake.

## Conclusion

We used an evidence-informed multifaceted approach to enhance a master's level CPG course for the needs of CPG developers and implementers in resource constrained settings. Dedicated, evidence-informed capacity development for CPG activities may empower current and future CPG developers and end-users and has the potential to positively influence CPG development and implementation.

### What is known about this topic

- High quality, evidence-informed CPGs offer a way of bridging the gap between policy, best-practice, local contexts and patient choice;
- There is an increasing need, and demand for, building capacity for evidence-informed CPG development and implementation.

### What this study adds

- There are limited robust evaluations of CPG capacity-development initiatives;
- This paper described the development, implementation and evaluation of a dedicated CPG training course designed to meet specific development needs in South Africa;
- The course fills a specific gap for decision-makers from resource constrained settings.

## Competing interests

The authors declare no competing interests.
